# Fuzheng Huayu recipe alleviates hepatic fibrosis via inhibiting TNF-α induced hepatocyte apoptosis

**DOI:** 10.1186/1472-6882-14-449

**Published:** 2014-11-18

**Authors:** Yan-yan Tao, Xiu-chuan Yan, Tao Zhou, Li Shen, Zu-long Liu, Cheng-hai Liu

**Affiliations:** Institute of Liver Diseases, Shuguang Hospital affiliated to Shanghai University of Traditional Chinese Medicine, 528 Zhangheng Road, Pudong New Area, Shanghai, 201203 China; Shanghai Key Laboratory of Traditional Chinese Clinical Medicine, Shanghai, 201203 China; Key Laboratory of Liver and Kidney Diseases (Shanghai University of Traditional Chinese Medicine), Ministry of Education, Shanghai, 201203 China; E-Institute of TCM Internal Medicine, Shanghai Municipal Education Commission, Shanghai, 201203 China

**Keywords:** Fuzheng Huayu recipe, Hepatocyte, Hepatic stellate cell, Apoptosis, Liver fibrosis

## Abstract

**Background:**

What was the relationship of Fuzheng Huayu recipe (FZHY) inhibiting hepatocyte apoptosis and HSC activation at different stage of liver fibrosis? In order to answer this question, the study was carried out to dynamically observe FZHY’s effect on hepatocyte apoptosis and HSC activation and further explored underling mechanism of FZHY against hepatocyte apoptosis.

**Methods:**

Mice were randomly divided into four groups: normal, model, FZHY, and N-acetylcystein (NAC) groups. Acute hepatic injury and liver fibrosis in mice were induced by CCl_4_. Three days before the first CCl_4_ injection, treatment with FZHY powder or NAC respectively was started. *In vitro*, primary hepatocytes were pretreated with FZHY medicated serum or Z-VAD-FMK and then incubated with ActD and TNF-α. Primary HSCs were treated with DNA from apoptotic hepatocytes incubated by Act D/TNF-α or FZHY medicated. Liver sections were analyzed for HE staining and immunohistochemical evaluation of apoptosis. Serum ALT and AST, Alb content and TNF-α expression in liver tissue were detected. Hyp content was assayed and collagen deposition was visualized. Expressions of α-SMA and type I collagen were analyzed by immunofluorescence and immunoblotting. Flow cytometry, immunofluorescence, and DNA ladder for hepatocyte apoptosis and immunoblotting for TNF-R1, Bcl-2 and Bax were also analyzed.

**Results:**

Mice showed characteristic features of massive hepatocytes apoptosis in early stage of liver injury and developed severe hepatic fibrosis in later phase. FZHY treatment significantly alleviated acute liver injury and hepatocyte apoptosis, and inhibited liver fibrosis by decreasing α-SMA expression and hepatic Hyp content. *In vitro*, primary hepatocytes were induced by TNF-α and Act D. The anti-apoptotic effect of FZHY was generated by reducing TNFR1 expression and balancing the expressions of Bcl-2 and Bax. Meanwhile, the nuclear DNA from apoptotic hepatocytes stimulated HSC activation in a dose dependent manner, and the DNA from apoptotic hepatocytes treated with FZHY or Z-VAD-FMK reduced HSC activation and type I collagen expression.

**Conclusion:**

These findings suggested that FZHY suppressed hepatocyte apoptosis through regulating mediators in death receptor and mitochondrial pathways, and the effect of FZHY on hepatocyte apoptosis might play an important role in inhibiting liver fibrosis.

## Background

Liver fibrosis is a wound healing process elicited by chronic liver injury [1]. Liver injuries, such as hepatocytic inflammatory necrosis and apoptosis, are the precursors of liver fibrosis [2]. Chronic liver injury of various etiologies results in hepatocyte apoptosis, and subsequent transdifferentiation of hepatic stellate cells (HSCs) into an activated myofibroblast (α-SMA-expressing) and thereby acquires fibrogenic properties producing extracellular matrix (ECM) proteins with an upregulation of profibrogenic cytokines such as TGF-β [1]. Hepatocyte apoptosis triggers HSC activation either directly by phagocytosis of the apoptotic bodies [3], or indirectly by damage-associated molecular patterns inducing the migration and activation of HSC [4]. Thus rational treatment approaches for liver fibrosis may include drugs that target hepatocyte apoptosis, HSC activation, or both.

Molecular mechanism of hepatic fibrosis has been elucidated much more clearly, however, progress in the treatment of liver fibrosis remains alarmingly slow [5, 6]. In the recent years, searching effective preparation against hepatic fibrosis from traditional chinese medicine (TCM) in China has achieved a big progress [7–9]. Fuzheng Huayu recipe (FZHY) is one of the well studied anti-fibrotic products in China. FZHY consists of six Chinese medicinal herbs [10], namely *Radix Salvia Miltiorrhizae* (Danshen)*, Cordyceps* (Chongcao), *Semen Persicae* (Taoren)*, Gynostemma Pentaphyllammak* (Jiaogulan), *Pollen Pini* (Songhuafen), *Fructus Schisandrae Chinensis* (Wuweizi). It has been approved by SFDA as a drug and widely used to treat hepatic fibrosis in China since 2002. And now its efficacy against liver fibrosis was confirmed in phase II clinical trial carried out in US (http://www.clinicaltrials.gov).

Previous studies have shown that FZHY exerts good effects against liver fibrosis in both animal experiments and clinical trials [11–14], the potential mechanisms of which are well studied and summarized in a recent published review [15]. Studies show that FZHY regulates many aspects of hepatic fibrosis, among them, inhibition of HSC activation is considered as a prominent effect [16]. It is widely known that hepatocyte apoptosis plays a pivotal role in hepatic fibrogenesis, and FZHY also shows a good effect on hepatic inflammation as well as hepatocyte apoptosis [17].While how the two mechanisms contributed to FZHY’s efficacy on liver fibrosis at different stage remained unclear. In this study, we carried out *in vivo and in vitro* studies on dynamic changes of hepatocyte apoptosis and HSC activation at different stage of liver fibrosis and effect of FZHY on hepatocyte apoptosis and HSC activation and further explored underling mechanism of FZHY against hepatocyte apoptosis in the early stage of liver fibrosis.

## Methods

### Reagents

Carbon tetrachloride (CCl_4_) was supplied by National Pharmaceutical Group Chemical Reagent Co., Ltd. (Shanghai, China). Actinomycin D (ActD) was purchased from AppliChem (Darmstadt, Germany). Tumor necrosis factor-α (TNF-α) and TACSTM Annexin V-fluorescein isothiocyanate (FITC) were obtained from R&D Systems (Minneapolis, MN, USA). Rat TNF alpha ELISA Kit was purchased from ThermoFisher Scientific Inc. In Situ Apoptosis Detection Kit (terminal deoxynucleotidyl transferase-mediated dUTP nick-end labeling, TUNEL) was from Chemicon International (Temecula, CA, USA). DNeasy Blood & Tissue Kit was provided by Qiagen (Hilden, Germany). The rabbit polyclonal antibody to TNF-α receptor type 1 (TNF-R1) (human), mouse monoclonal antibody to Bax (mouse) were purchased from Santa Cruz Biotechnology (Santa Cruz, CA, USA). The rabbit monoclonal antibody to Bcl-2 was from Cell Signaling Technology (Beverly, MA, USA). Pronase E and DNase were obtained from Roche (Switzerland). Collagenase type IV was from Sigma (St. Louis, MO). Medium 199 (M199) and minimum essential medium eagle w/o Ca^2+^ (MEM) were from Gibco. OptiPrep™ was from Axis-shield, Norway. N-acetylcystein (NAC) and Z-VAD-FMK were from Sigma. All other chemicals used in the experiment were of analytical grade.

### Drugs

Fuzheng Huayu recipe (FZHY) was provided by Shanghai Sundise Medicine Technology Development Co., Ltd., China (SFDA approval No: Z20050546) (Shanghai, China). Its formula, extraction process and chemical fingerprint have been described in detail [10].

### Animals

C57BL/6 (B6) mice (8 weeks of age, with average body weight of 23 ± 2 g) were purchased from the Experimental Animal Center, Chinese Academy of Science (Shanghai, China). All mice were housed in a specific pathogen-free and controlled environment. All animal studies were performed according to the Guide for the Care and Use of Laboratory Animals of the National Institutes of Health. The protocol was approved by the Committee on the Ethics of Animal Experiments of Shanghai University of Traditional Chinese Medicine, China.

### Experimental protocol

**Figure 1:**
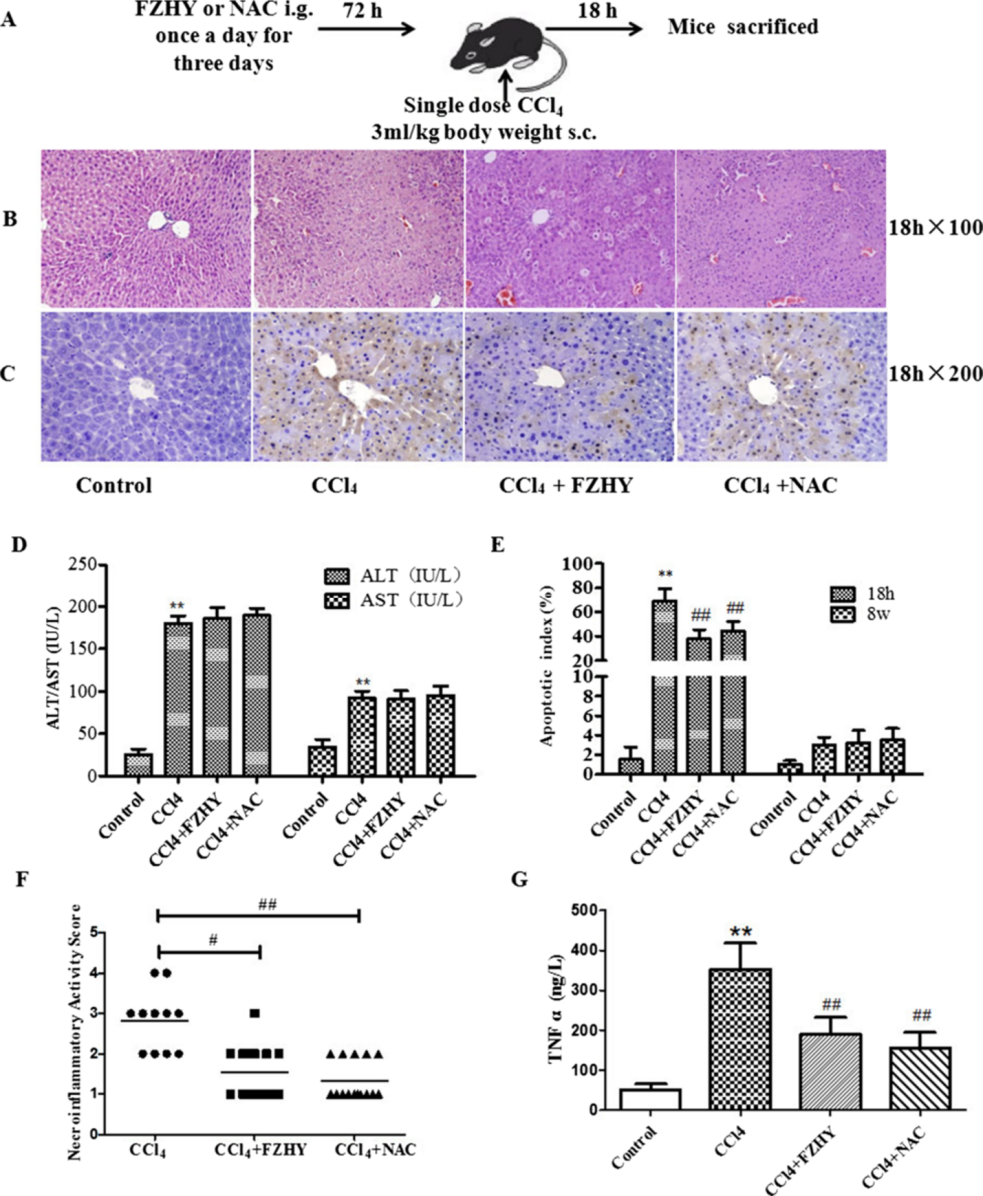
**Fuzheng Huayu recipe (FZHY) and N-acetylcysteine (NAC) attenuates hepatocyte apoptosis induced by CCl**_**4**_ **.** Mice were respectively treated orally with FZHY (4.0 g/kg) or NAC (0.1 g/kg) daily for 3 d. Then, mice were subcutaneously injected with 100% CCl_4_ for 18 hours to develop acute liver injury **(A)**. Liver sections were subjected to either hematoxylin-eosin staining to detect necrosis and inflammatory cell infiltration (**B**, × 100) or to the terminal deoxynucleotidyl transferase-mediated dUTP nick-end labeling (TUNEL) assay to detect cell apoptosis (**C**, × 200). **(D)** Serum ALT and AST levels in the four groups (Control group, n = 10; CCl_4_ treated, n = 11; FZHY treated, n = 15; NAC treated, n = 15). **(E)** The apoptotic index (AI) of five high-power fields at × 400 magnification in each tissue specimen. **(F)** Necrosis and inflammatory cell infiltration scores in the four groups (Control group, n = 10; CCl_4_ treated, n = 11; FZHY treated, n = 15; NAC treated, n = 15). **(G)** Quantitative ELISA analysis of hepatic TNF α expression in the four groups (Control group, n = 10; CCl_4_ treated, n = 11; FZHY treated, n = 15; NAC treated, n = 15). ^*^ *P* < 0.05, ^**^ *P* < 0.01 *vs.* control group; ^#^ *P* < 0.05, ^##^ *P* < 0.01 *vs.* CCl_4_ group.

**Figure 2:**
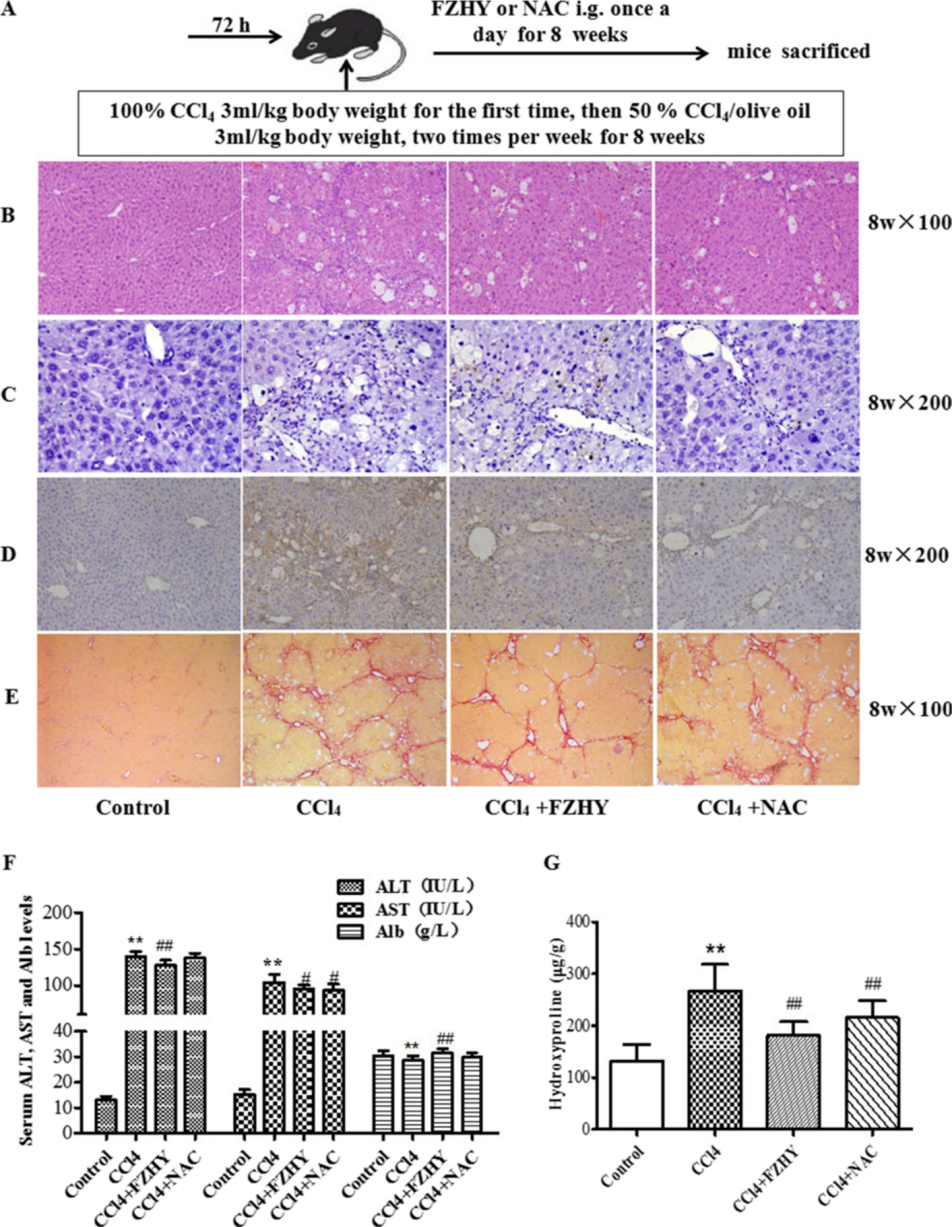
**Fuzheng Huayu recipe (FZHY) and N-acetylcysteine (NAC) improves hepatic fibrosis.** Mice were respectively treated orally with FZHY (4.0 g/kg) or NAC (0.1 g/kg) daily for 3 d before CCl_4_ treatment. Then, mice were injected at 8 weeks of age with 100% CCl_4_ 3 ml/kg body weight for the first time, then 50% CCl_4_ /olive oil 3 ml/kg body weight, two times per week for 8 weeks to develop hepatic fibrosis **(A)**. Liver sections were subjected to either hematoxylin-eosin staining to detect inflammatory cell infiltration and necrosis (**B**, × 100) or to the terminal deoxynucleotidyl transferase-mediated dUTP nick-end labeling (TUNEL) assay to detect cell apoptosis (**C**, × 200) or to immunohistochemical staining for α-SMA for detection of activated hepatic stellate cells (HSCs) (**D**, × 200) or to Sirius Red staining for detection collagen deposition (**E**, × 200). **(F)** Serum ALT and AST levels and Alb content in the four groups (Control, n = 10; CCl_4_ treated, n = 11; FZHY treated, n = 11; NAC treated, n = 12). **(G)** Hepatic Hyp content was determined using Jamall’s method. The Hyp content was increased significantly in CCl_4_ treated group compared with the control group. FZHY or NAC treatment significantly decreased liver Hyp content. ^**^ *P* < 0.01 *vs.* control group; ^##^ *P* < 0.01 *vs.* model.

**Figure 3:**
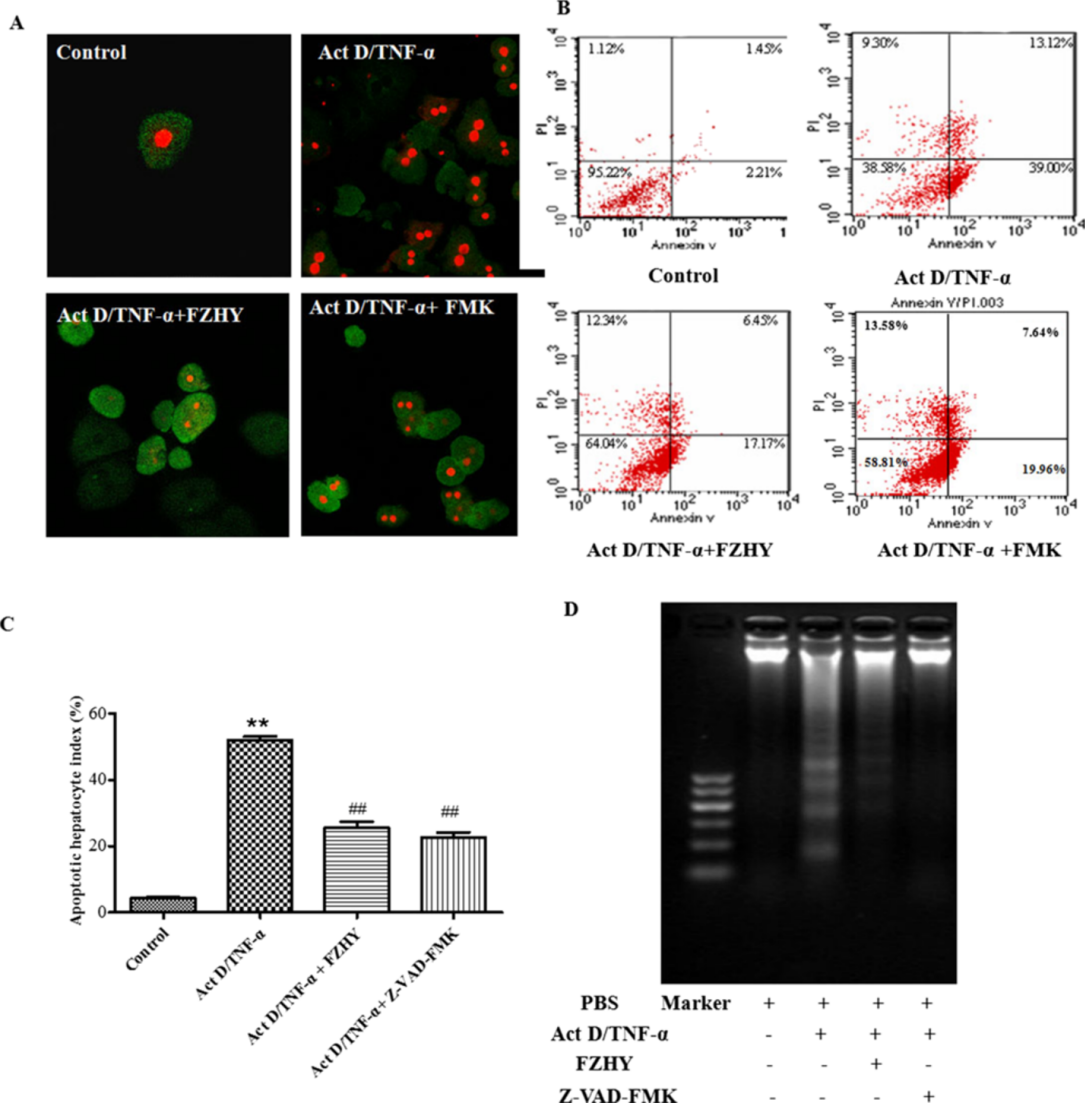
**Fuzheng Huayu recipe (FZHY) inhibits Act D/TNF-α induced hepatocyte apoptosis.** Z-VAD-FMK (FMK) is a synthetic peptide that irreversibly inhibits activity of caspase family proteases and blocks apoptosis, and was used as a positive control in this work. Twenty four hours after plating, primary hepatocytes were pretreated with 10% FZHY medicated serum or 10% rats’ serum plus 50 μM Z-VAD-FMK for 18 h and then incubated with 200 ng/ml ActD and 20 ng/ml TNF-α for 6 h. **(A)** Confocal microscopy observation of hepatocyte apoptosis stained by FITC labeled Annexin V (green) and propidium iodide (PI) (red) (×400). **(B)** Cells were trypsinized and stained with Annexin V and PI followed by analysis with flow cytometry. Early apoptotic cells (Annexin V positive and PI negative) were in the right lower quadrant. Late apoptotic or necrotic cells were in the right upper quadrant. **(C)** The apoptotic index (AI) of five high-power fields at × 400 magnification of confocal microscopic figures in Figure 3A. ^**^ *P* < 0.01 *vs.* control group; ^##^ *P* < 0.01 *vs.* Act D/TNF-α treated group. **(D)** Primary hepatocytes were pretreated with 10% FZHY medicated resum or 10% rats’ serum + 50 μM Z-VAD-FMK for 18 h and then incubated with 200 ng/ml ActD and 20 ng/ml TNF-α for 6 h. Cells were harvested, and DNA was purified by the DNeasy Kit. DNA was separated on 1.5% agarose gel electrophoresis and visualized under ultraviolet light. Lane 1, marker; lane 2, phosphate-buffered saline (PBS) treated cells; lane 3, PBS and Act D/TNF-α treated cells; lane 4, FZHY and Act D/TNF-α treated cells; and lane 5, Z-VAD-FMK and Act D/TNF-α treated cells. Agarose gel electrophoresis showed that FZHY and Z-VAD-FMK inhibited apoptosis of hepatocytes induced by Act D and TNF-α.

**Figure 4:**
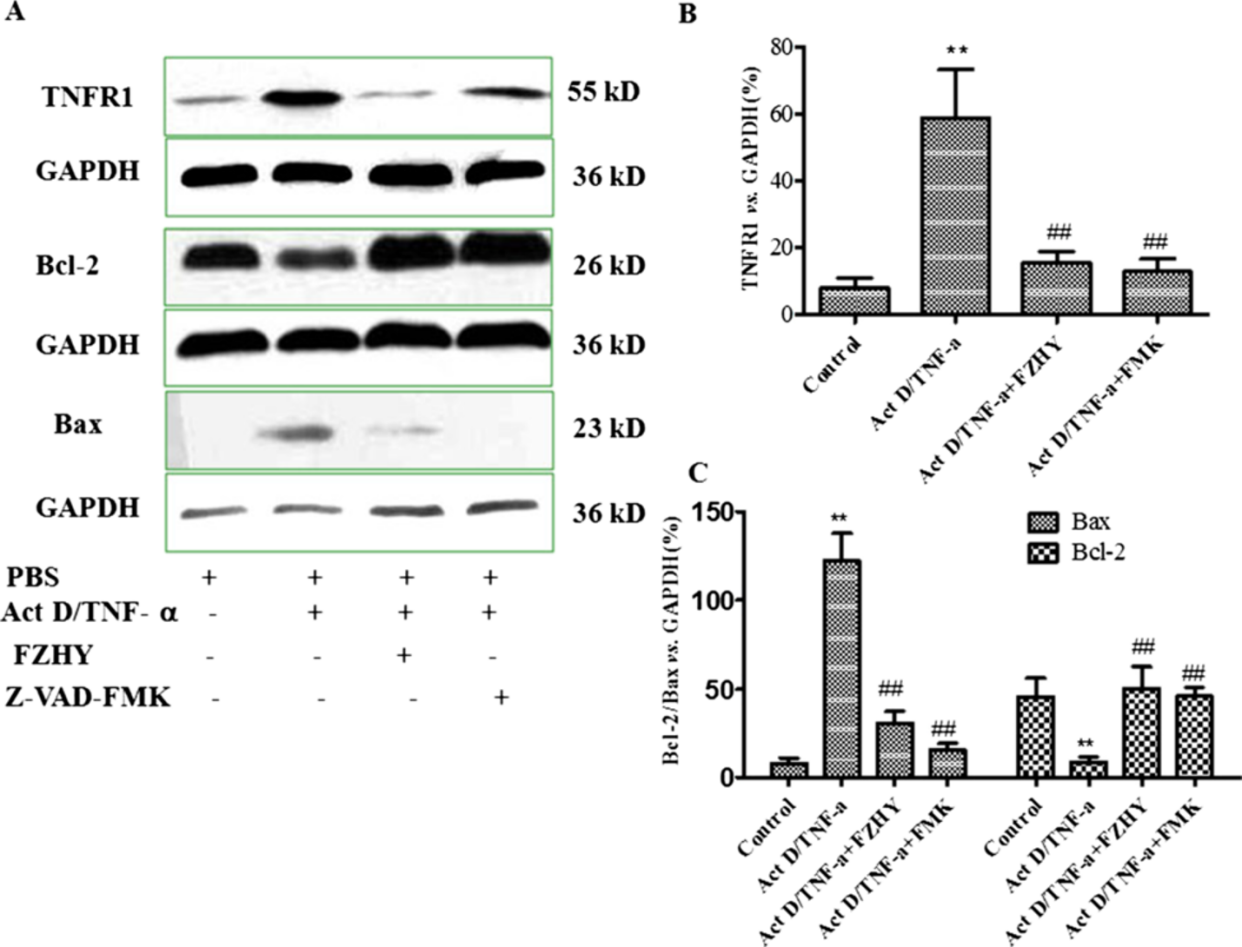
**Fuzheng Huayu recipe (FZHY) inhibits Act D/TNF-α induced hepatocyte apoptosis via regulating the apoptotic and anti-apoptotic proteins in mitochondria.** Primary hepatocytes were pretreated with 10% FZHY medicated resum or 10% rats’ serum plus 50 μM Z-VAD-FMK for 18 h and then incubated with 200 ng/ml ActD and 20 ng/ml TNF-α for 6 h. **(A)** Western blot analysis of TNFR1, Bcl-2 and Bax in hepatocytes. TNFR1 expression was low in untreated hepatocytes, whereas TNFR1 expression increased after ActD and TNF-α stimulation. The expression of TNFR1 was significantly down-regulated by FZHY or Z-VAD-FMK treatment. Similarly, the expressions of Bcl-2 and Bax were balanced by FZHY or Z-VAD-FMK treatment. Graphic presentation of the relative expressions of TNFR1 **(B)** and Bcl-2 and Bax **(C)**. Values were represented as the density of TNFR1, Bcl-2 or Bax *vs.* housekeeping gene GAPDH (%) from 3 samples. ^**^ *P* < 0.01 *vs.* control; ^##^ *P* < 0.01 *vs.* Act D/TNF-α treated group.

**Figure 5:**
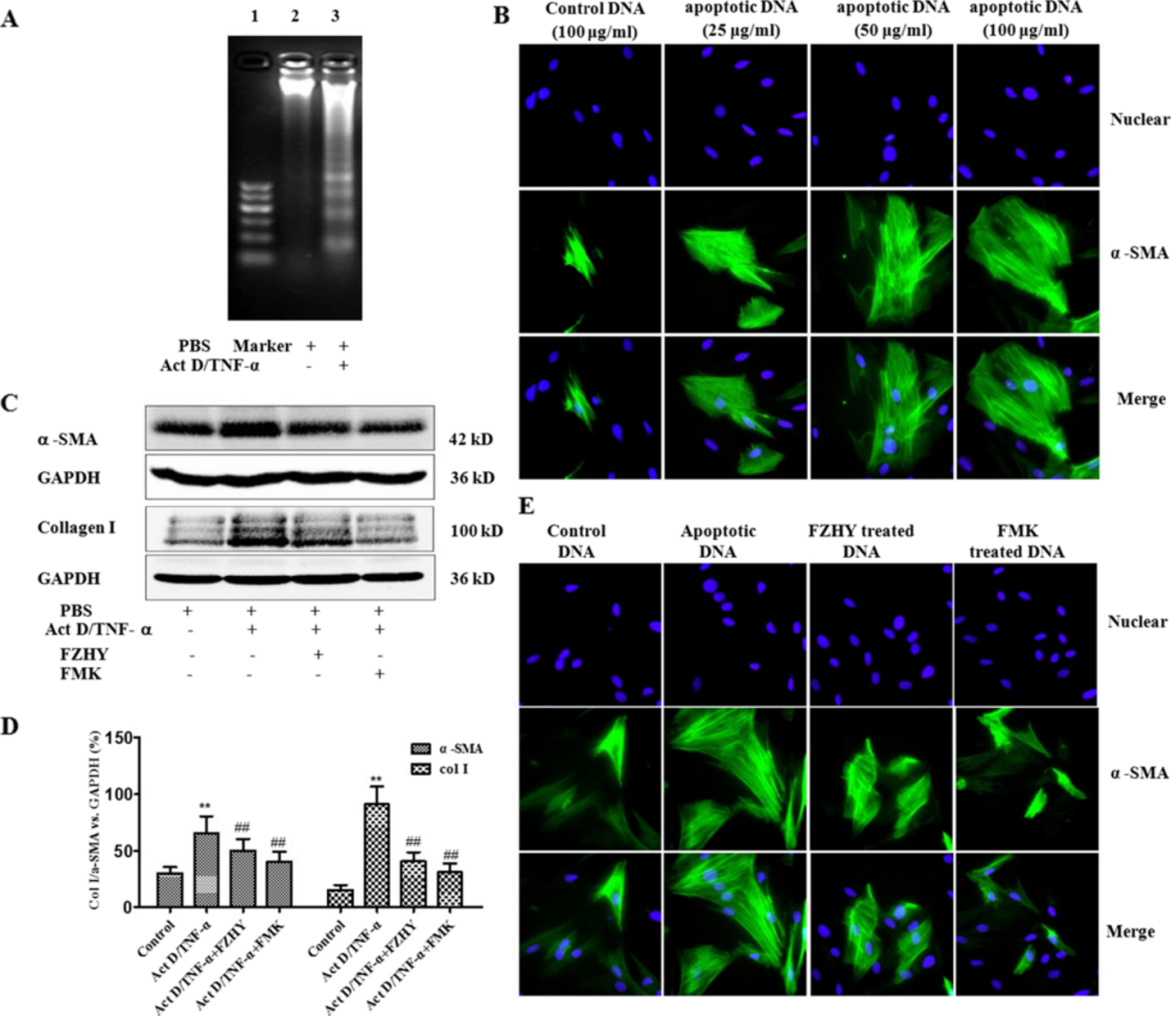
**The DNA of Apoptotic hepatocyte up-regulates α-SMA expression in rat HSCs and FZHY inhibited hepatocyte apoptosis and the expressions of α-SMA and type I collagen in HSCs. (A)** Fragmented apoptotic DNA in cultured hepatocytes was induced by 200 ng/ml Act D and 20 ng/ml TNF-α. Lane 1, Marker, Lane 2; phosphate-buffered saline (PBS)-treated hepatocytes; Lane 3, Act D/TNF-α treated hepatocytes. **(B)** Primary hepatocytes were incubated with or without 200 ng/ml ActD and 20 ng/ml TNF-α for 6 h. 100 μg/ml DNA from hepatocytes incubated with PBS acted as a control. 25 ~ 100 μg/ml DNA from apoptotic hepatocyte stimulated α-SMA expression in rat HSCs in a dose dependent manner (Immunofluorescence staining, × 400). **(C)** Western blot analysis of expressions of α-SMA and type I collagen in rat HSCs. **(D)** Graphic presentation of the relative expressions of α-SMA and type I collagen. The values were represented as the density of α-SMA and collagen I *vs.* housekeeping gene GAPDH (%) from 3 samples. **(E)** Primary hepatocytes were incubated with or without 200 ng/ml ActD and 20 ng/ml TNF-α for 6 h. Then, 100 μg/ml DNA from apoptotic hepatocytes was added into HSCs after culture for 4 days. 100 μg/ml DNA from hepatocytes incubated with PBS acted as a control. The apoptotic DNA from hepatocytes treated by FZHY or Z-VAD-FMK (FMK) had less stimulating effects on α-SMA expression in HSCs (Immunofluorescence staining, × 400). ^**^ *P* < 0.01 *vs.* Control; ^##^ *P* < 0.01 *vscpc* Act D/TNF-α group.

Four experimental protocols were followed. Firstly, to address the influence of FHZY on apoptosis of hepatocyte following CCl_4_-induced acute hepatic injury. Mice were subcutaneously injected with 100% CCl_4_ 3 ml/kg body weight for one time, starting at 8 weeks of age. Three days before the first CCl_4_ injection, once-daily treatment with FZHY powder 4.0 g (crude drug)/kg or NAC 0.1 g/kg body weight respectively was started and mice were followed as noted in Figure 1. Eighteen hours after the first CCl_4_ injection, mice were analyzed for liver function, hematoxylin-eosin (HE) staining and immunohistochemical evaluation of apoptosis (Figure 1). Secondly, to focus on the effect of FHZY on established liver fibrosis, mice were injected at 8 weeks of age with 100% CCl_4_ 3 ml/kg body weight for the first time, then 50% CCl_4_/olive oil 3 ml/kg body weight, two times per week for 8 weeks. Three days before the first CCl_4_ injection, once-daily treatment with FZHY powder 4.0 g (crude drug)/kg or NAC 0.1 g/kg body weight respectively was started and mice were followed as noted in Figure 2. At 16 weeks of age, or 8 weeks following the initial exposure to CCl_4_, mice were sacrificed for histological assessment of liver fibrosis, and immunohistochemical evaluation of apoptosis (Figure 2). Thirdly, to detect the effect of FHZY on hepatocyte apoptosis induced by Act D/TNF-α (Figure 3), After cultured for 24 hour, primary hepatocytes were treated with 10% FZHY-medicated serum or 10% rats’ serum + 50 μM Z-VAD-FMK for 18 h and then incubated with 200 ng/ml ActD and 20 ng/ml TNF-α for another 6 h. Immunofluorescence, flow cytometry and DNA ladder for hepatocyte apoptosis and immunoblotting for TNFR1, Bcl-2 and Bax were also analyzed. Finally, to explore whether FZHY attenuated hepatic fibrosis by inhibiting apoptosis of hepatocytes, we performed the subsequently experiments. At day 4 after isolation, primary HSCs were treated with the DNA of apoptotic hepatocytes incubated by Act D/TNF-α and 10% FZHY-medicated serum. Immunofluorescence and immunoblotting for α-SMA and type I collagen in HSCs were analyzed (Figures 4 and 5).

### Histological and immunohistological analysis of liver sections

Liver tissues were fixed with 10% formalin, embedded in paraffin and cut into 4-μm sections stained with hematoxylin-eosin (H&E). Necroinflammatory activity in hepatic tissue was graded by a “blinded” liver pathologist according to the Scheuer scoring system [18]. For quantitative assessment of fibrosis, sections were stained with Sirius Red for quantitative analysis of collagen content [19]. α-SMA, a marker of hepatic stellate cells activation, was assessed by staining with rabbit anti-α-SMA antibody (clone 1A4; Dako, Glostrup, Denmark) and visualized with HRP labeled anti-rabbit antibody (ChemMate™ EnVision™ Detection Kit, Dako).

### Analysis of serum transaminase activity and Alb level

The activities of serum alanine aminotransferase (ALT) and aspertate aminotransferase (AST) and serum albumin (Alb) level were quantitated by following the instructions provided by the manufacturer, including use of standardization [20].

### Hydroxyproline assay

Hyp content of liver was assayed with HCl hydrolysis according to a modification of method by Jamall et al. [21]. The Hyp content of the liver as an indirect measure of tissue collagen content was expressed as microgram per gram of liver weight (μg/g).

### TUNEL assay

For detection of cell apoptosis, TdT-mediated dUTP nick end labeling (TUNEL) analysis was performed as previously described [22]. In each tissue specimen, five high-power fields at × 400 magnification were randomly selected and the apoptotic index (AI) was calculated as the percentage of positive cells, using the equation: AI = (number of positive cells/total number of cells) × 100% [23].

### Preparation of FZHY-medicated serum

FZHY powder was administered to the rats at a dose of 2 g (crude drug)/kg body weight by intragastic gavage twice a day for 3 days. The rats were given FZHY 1 h before being sacrificed on the 4 th day. Blood samples were collected under anesthesia. The samples were placed at 4°C for 4 h and then centrifuged at 3000 rpm for 20 min. The sera were inactivated at 56°C for 30 min and then stored at −20°C for further use.

### Primary hepatocytes and HSCs isolation and culture

Primary hepatocytes isolation. Male C57BL/6 mice mentioned above were used in this study. Primary hepatocytes were isolated using a two-step in situ collagenase perfusion method. Briefly, the hepatic portal vein was canulated in situ, then it was perfused with calcium and magnesium-free Hanks’ salt solution for 10 minutes, followed by 0.45–0.5 mg/mL of type IV collagenase solution at 37°C until the liver capsule was incised. After perfusion, the thick fibrous connective tissue was discarded and filtered cell suspensions were harvested. Primary hepatocytes were then collected by centrifugation and seeded in M 199 containing 10% FBS. The viability of the freshly isolated hepatocytes was determined by trypan blue exclusion and cell samples with viability greater than 90% were used in the subsequent assays.

Primary HSCs isolation. Sprague–Dawley rats (with average body weight of 400 ± 30 g) were used in this study. Primary HSCs were isolated from normal rat liver by a two step pronase-collagenase perfusion and single-step density gradient of OptiPrep™. Briefly, the liver were perfused firstly with calcium and magnesium - free solution for 10 min at 37°C, and then with Pronase E solution (2.4 mg/ml) and collagenase IV solution (0.3–0.45 mg/ml) for 10 min and 30 min respectively at 37°C, the digested liver were excised, dispersed in serum-free MEM,and filtered through gauze. The cells were mixed with 12.25 ml Gey’s balanced salt solution (GBSS: Dissolve the following in 500 ml water: 7.0 g NaCl, 0.37 g KCl, 70 mg MgSO_4_.7H_2_O, 220 mg) and 2.75 ml of 60% (w/v) of OptiPrep™. The gradient was generated by placing 10 ml of GBSS on top of the liver cell mixture in a 50-ml centrifugation tube. After centrifugation (1,400 g, 20°C, 20 min) the cells were aspirated from above the interface, washed once in serum-free MEM and subsequently cultured under an atmosphere of 5% CO_2_, 95% air in 100-mm tissue culture dishes using M199 containing penicillin G 100 U/ml, streptomycin sulfate 100 U/ml and 10% FBS. For immunofluorescent stain, cells were cultured on 12-well plate. Cell viability was greater than 90% as assessed by trypan blue exclusion. Purity was 90–95% as assessed by immunoflurescent stain of Desmin.

### Hepatocyte apoptosis determined by confocal microscopy and flow cytometry analysis

Primary hepatocytes cultured on cover slips were washed with cold PBS twice and incubated with FITC-labeled Annexin V and propidium iodide (PI) for 2 min. After washing twice with the binding buffer provided by the manufacture, the cells were mounted with fluorecscence compatible medium. View images immediately by confocal microscopy (TCS-SP2, Leica, Mannheim, Germany).

Primary hepatocytes in each group were harvested, washed with cold PBS, and stained with Annexin V and PI for 15 min in accordance with the manufacturer’s instructions. The analysis was performed with a FACScan flow cytometer (BD Biosciences, San Jose, CA, USA) using the Cell Quest software (BD Biosciences).

### Preparation of apoptotic DNA from hepatocyte

*In vitro* experiment were performed referred to Watanabe [4]. Primary hepatocytes were cultured in a 60 mm dish, and when near confluent were exposed to 200 ng/ml ActD and 20 ng/ml TNF-α referred to our previous work. Cell apoptosis was evident 6 hours after exposure. At this time, DNA was extracted by kits following the manufacturer’s instructions. DNA was quantified and apoptosis was confirmed by running the extracted DNA on an agarose gel to visualize the characteristic laddering of fragmented DNA.

### Immunofluorescence for α -SMA

Indirect fluorescence immunostaining of α-SMA was performed. Briefly, HSCs cultured on cover slips were washed with cold PBS twice, fixed with 4% polyformaldehyde in PBS at room temperature for 15 min, and permeabilized with 0.1% Triton X-100 in 0.1% sodium citrate at room temperature for 15 min. The cells were blocked with 5% bovine serum albumin in PBS buffer for 30 min at room temperature and then incubated overnight at 4°C with primary mouse monoclonal antibody to mouse α-SMA (1:100) (Sigma, USA). Then cells were stained with Alexa Fluor® 488 goat anti-mouse IgG (H + L) (1:100) (Invitrogen, USA). After washing, cells were stained with Hoechst 33258 to visualize the nuclei. Stained cells were mounted with antifade mounting medium and viewed under fluorescence microscope (Olympus, Japan).

### Enzyme-linked immunosorbent assay for TNF α

The expression of TNF-α protein in the supernatant of rat liver homogenate was determined using an Enzyme-linked immunosorbent assay from ThermoFisher Scientific Inc. according to the manufacturer’s protocol.

### Western blot analysis

Cells were homogenized in RIPA buffer (50 mmol/L Tris–HCl, 150 mmol/L NaCl, 1 mmol/L PMSF, 1 mmol/L EDTA, 1% Triton X-100, 1% sodium deoxycholate, 0.1% SDS, pH 7.4), and the homogenate was centrifuged at 12,000 g for 30 min. The protein concentrations of supernatant were determined by the BCA protein assay. All procedures were performed at 4°C. The solubilized cell samples, each containing 30 μg of protein, were subjected to 10% SDS-PAGE gel electrophoresis in reducing and denaturing conditions and then transferred onto nitrocellulose membrane. The membranes were then blocked with 5% nonfat milk in Tris- buffered saline containing 0.1% Tween (TBST, 100 mmol/L Tris–HCl, pH 8.0, 150 mmol/L NaCl, and 0.1% Tween 20) for 1 h at room temperature, followed by incubation with primary antibodies overnight at 4°C as follows: Rabbit polyclonal antibody to human TNF-R1 (1:200), Rabbit monoclonal antibody to Bcl-2 (1:1000), Mouse monoclonal antibody to mouse Bax (1:200), and GAPDH (Kangcheng, China), as housekeeping control. Thereafter, the blots were washed with TBS-T 3 times, followed by incubation with the second antibody (anti-mouse or anti-rabbit horseradish peroxidase-conjugated antibody, Santa Cruz) for 1 h at room temperature. After washing, the blots were developed with SuperSignal West Pico Chemiluminescent Substrate (Thermo Scientific, USA) and then exposed to X-ray films (Kodak, China) in accordance with the manufacturer’s protocol.

### Statistical analysis

Data are expressed as mean ± SD. Data were analyzed using a one-way analysis of variance (ANOVA) as well as the LSD test, and P < 0.05 was considered statistically significant.

## Results

### FZHY attenuates hepatocyte apoptosis in CCl_4_ induced mice

In a recent study, we induced hepatocyte apoptosis *in vivo* through injection of Lipopolysaccharide (LPS)/D-galactosamine (GalN) in mice [17]. FZHY treatment significantly attenuated hepatocyte apoptosis as indicated by TUNEL staining. FZHY exerted both anti-fibrotic and anti-apoptotic functions, but whether the effect of FZHY on liver fibrosis was primarily due to its inhibitory effect on hepatocyte apoptosis. Here in order to make this clear, we first studied acute liver injury by subcutaneously injecting CCl_4_ for one time and treating daily with FZHY or NAC (Figure 1A). Liver sections revealed less necrosis and inflammatory cells infiltration were seen in FZHY or NAC-treated mice compared with CCl_4_ treated mice (*P* < 0.05) (Figure 1B & F). FZHY or NAC treatment markedly down-regulated hepatocyte apoptosis in the liver of mice injected with CCl_4_ (*P* < 0.05) (Figure 1C). As shown in Figure 1D, there were no significant differences in ALT and AST levels among FZHY-treated group, NAC-treated group and CCl_4_ treated group (*P* >0.05). Then, the concentration of TNF-α in liver tissue was detected by ELISA. TNF-α level was very low in normal liver. After a single dose of CCl_4_ treatment, the expression of TNF-α protein was significantly increased, and FZHY or NAC treatment attenuated TNF-α expression in CCl_4_ treated mice (*P* < 0.05) (Figure 1G). Quantitation of the apoptotic hepatocytes revealed that FZHY or NAC treatment significantly reduced hepatocyte apoptosis in CCl_4_-induced acute liver injury in mice (*P* < 0.05) (Figure 1E).

### FZHY alleviates liver injury and hepatic fibrosis induced by CCl_4_ in mice

We next studied CCl_4_-induced liver fibrosis by subcutaneously injecting CCl_4_ for 8 weeks and treating daily with FZHY or NAC (Figure 2A). Liver sections revealed less vacuolated cells and significantly improved portal inflammation in the FZHY or NAC treated mice compared to controls (Figure 2B). As shown in Figure 2F, ALT and AST levels were slightly lower and Alb content was higher in FZHY-treated mice compared with control (*P* < 0.05). TUNEL assay revealed that apoptotic cells were not significantly different between FZHY or NAC treated and untreated mice with or without CCl_4_ (Figures 2C and 1E). Sirius red staining showed that subcutaneous injection of CCl_4_ in mice for 8 weeks developed collagen deposition, false lobules formation. Notable reduction of the thickening of the collagen bundles was seen in FZHY or NAC-treated mice compared with control (Figure 2E). Immunohistochemical analysis revealed that the expression of α-SMA was low in normal liver, whereas it was prominent in mice receiving CCl_4_ injection, and FZHY or NAC treatment attenuated α-SMA expression (Figure 2D). To evaluate the progression of fibrosis in a quantitative manner, hepatic hydroxyproline (Hyp) content was measured. Liver collagen content, expressed as microgram (μg) of Hyp/gram (g) of liver tissue, was shown in Figure 2G. Hyp content in the CCl_4_-treated mice was approximately 202% of that of the control group (*P* < 0.01), suggesting abundant accumulation of collagen in CCl_4_ induced mice. It was consistent with the observation of marked fibrosis and accumulation of collagen bundles in CCl_4_-induced mice by histopathological examination. There was a significant decrease (*P* < 0.01) in liver Hyp content in FZHY or NAC treated mice, suggesting that FZHY or NAC ameliorated hepatic collagen deposition in CCl_4_-induced liver injury.

### FZHY attenuates hepatocyte apoptosis by regulating apoptotic mitochondria pathway

To detect the effect of FZHY on hepatocyte apoptosis, primary hepatocytes were incubated with Act D/TNF-α for 6 hours (Figure 3). The cleavage of chromosomal DNA into fragments is a biochemical hallmark of apoptosis. Although it has been documented that hepatic apoptosis is not always associated with DNA fragmentation [24], typical DNA ladder was shown with agarose gel electrophoresis which provided additional evidence in support of cell apoptosis after exposure to Act D/TNF-α (Figure 3). Confocal microscopy showed that few apoptotic hepatocytes were seen in control group, while a large number of hepatocytes developed apoptosis after 6 hours incubation with Act D and TNF-α, FZHY treatment significantly reduced the amount of apoptotic hepatocytes (*P* < 0.01) (Figure 3A and C). Similarly, as shown in Figure 3B with flow cytometry, only 3.6% of hepatocyte apoptosis was seen in the control group, while approximately 50% of hepatocyte developed early or late apoptosis after induction by Act D and TNF-α, FZHY or Z-VAD-FMK treatment reduced 30% of hepatocyte apoptosis. As shown in Figure 3D, the appearance of typical DNA ladder determined by gel electrophoresis confirmed the existence of apoptotic hepatocyte after 6 hours exposure to ActD and TNF-α. FZHY or Z-VAD-FMK treatment markedly reduced hepatocyte apoptosis incubated by ActD/TNF-α. TNF-α signaling in hepatocyte apoptosis was well reviewed in many documents [25, 26]. Here the mitochondria pathway was selected to observe whether FZHY regulation was involved. Western blot analysis showed that FZHY or Z-VAD-FMK treatment significantly down-regulated TNFR1 expression compared with controls (*P* < 0.05) (Figure 4A and B). Similarly, the expressions of Bcl-2 and Bax were balanced by FZHY or Z-VAD-FMK treatment (*P* < 0.01) (Figure 4A and 4C).

### The DNA from TNF-α/Act D treated hepatocytes stimulates HSCs activation

Since FZHY exerted beneficial effects on ameliorating hepatocyte apoptosis and hepatic fibrosis in CCl_4_-injected mice, we were eager to know whether or not the anti-hepatocytic apoptosis-effect of FZHY was one of its mechanisms against liver fibrosis. To answer this question, we carried out the *in vitro* experiment that the DNA from TNF-α/Act D treated hepatocytes was added into primary isolated HSCs at day 4 after plating. As shown in Figure 5A, the appearance of typical DNA ladder determined by gel electrophoresis confirmed the existence of apoptotic hepatocyte DNA after 6 hours exposure to ActD and TNF-α. After co-incubation with 25 ~ 100 μg/ml apoptotic DNA fragmentation for 24 hours, the expressions of α-SMA in HSCs were unregulated in a dose dependent manner (Figure 5B), which indicated apoptotic hepatocyte DNA stimulated HSCs activation.

### FZHY-treated hepatocyte apoptotic DNA inhibits HSCs activation

However, it was not clear whether FZHY inhibiting HSCs activation correlated with its inhibiting hepatocyte apoptosis. Then, primary HSCs at day 4 after plating were incubated with 100 μg/ml purified DNA from each group. Immunofluorescence and western blotting analysis revealed that the expression of α-SMA was weak in HSCs incubated with 100 μg/ml control DNA, whereas it was prominent in HSCs incubated with 100 μg/ml apoptotic DNA. Consistent with the induced α-SMA expression, the expression of collagen I was markedly increased in HSCs incubated with 100 μg/ml apoptotic DNA compared with that incubated with control DNA. The apoptotic DNA of hepatocytes treatment with FZHY or VAD-FMK attenuated the expressions of α-SMA and type I collagen in HSCs (*P* < 0.01) (Figure 5C and D). The results indicated that FZHY inhibiting HSCs activation correlated with its inhibiting hepatocyte apoptosis.

## Discussion

Apoptosis is an active form of cell death that involves programmed cellular machineries leading to a progressive self-destruction of the cell. In contrary to necrosis, it can affect individual cells within a cell population. It is characterized by chronological alteration of intracellular biochemical signaling pathways followed by cellular morphological changes, DNA fragmentation, perturbation of mitochondrial membrane function and changes in the plasma membrane [27]. Hepatocyte apoptosis, one of the ubiquitous features of chronic and acute liver injuries, is reported to associate with liver fibrosis tightly. Proapoptotic stimulus induces hepatocyte apoptosis, meanwhile, the apoptotic cells release lipid signals for their uptake by kupffer cells and HSCs. Engulfment of the apoptotic bodies by HSCs and kupffer cells enhances their expression of pro-fibrogenic genes and death ligands (e.g., FasL). Persistent activation of HSCs and kupffer cells promotes further hepatocyte apoptosis, which culminates in hepatic inflammation, with generation of CXC chemokines (interleukin-8, macrophage inflammatory protein-2, et al.) and further HSC activation and liver fibrosis in a feed-forward-loop process [28].

The growing panoply of interactions between hepatocyte apoptosis, inflammatory, and fibrotic responses has therapeutic implications. For example, small molecule caspase inhibitors currently are being developed for clinical use [29–31], which could reduce hepatocyte apoptosis thereby attenuating inflammation, reducing HSC activation, and decreasing fibrosis. Acute and chronic liver injury result in increased local and systemic concentrations of transforming growth factor-beta 1 (TGF-β1) [32], a potent fibrogenic cytokine. N-acetylcysteine (NAC) is an antioxidant, a precursor of reduced glutathione, and an inhibitor of the profibrotic cytokine TGF-β1. NAC has been shown to prevent damage to DNA and proteins caused by mutagens and carcinogens and diminish apoptosis. Another study has suggested that NAC prevents experimental cirrhosis by two mechanisms: by preventing oxidative stress and by downregulating the profibrogenic cytokine TGF-β1 [33]. NAC is used as a control to prevent apoptosis *in vivo* study, which is quite popular for its ability to minimize oxidative stress and the downstream negative effects thought to be associated with oxidative stress. Carbon tetrachloride (CCl_4_) -induced liver injury is characterized by oxidative stress and fibrosis. In our current study, mice injected with CCl_4_ showed a characteristic of massive hepatocyte apoptosis in early stage of liver injury (Figure 1) and severe hepatic fibrosis in later stage (Figure 2). In this work we found that serum ALT and AST levels increased, the expression of TNF-α protein in liver tissue was significantly increased, hepatic parenchyma was distorted and vacuolar degeneration and necrosis of the hepatocytes (visualized by H&E stain) and hepatocyte apoptosis (detected by TUNEL assay) increased notably in acute liver injury induced by CCl_4_ intoxication. The results showed that FZHY and NAC effectively attenuated CCl_4_-induced acute liver injury and hepatocyte apoptosis (Figure 1). After 8 weeks of CCl_4_ intoxication, mice developed severe hepatic fibrosis, with obvious fibrotic septa and α-SMA expression, diminished apoptotic response and increased Hyp content. Administration of FZHY and NAC reduced collagen accumulation as evidence by marked reduction of Hyp content (Figure 2). This might be partly attributed to FZHY protecting hepatocytes from apoptosis in early stage of liver injury.

Excessive hepatocyte apoptosis is thought to lead to liver dysfunction and damage in a variety of liver diseases, and this process may occur during the initiation and/or progression of disease. Our previous work showed FZHY could protect hepatocytes from apoptosis and necrosis in acute liver injury induced by lipopolysaccharide (LPS)/D-galactosamine (D-GalN), and the mechanisms in part were associated with regulations of apoptotic factors—Bcl-2 and Bax [17]. However, whether or not FZHY affected, as well as how it might affect, the apoptotic signaling in primary hepatocytes were unknown. In subsequent experiments *in vitro*, primary mouse hepatocytes were used, and cells were stimulated by Act D plus TNF-α. Z-VAD-FMK, an irreversible general caspase inhibitor that irreversibly binds to the catalytic site of caspase proteases [34], was used as a control to prevent apoptosis *in vitro* study. In this study, we initially observed that a large number of hepatocytes developed apoptosis after 6 hours incubation with Act D plus TNF-α. Various methodologies were then used to assess the effects of FZHY on hepatocytes apoptosis, including flow cytometry, DNA electrophoresis and Annexin V and PI staining. Our results confirmed that FZHY had the same effect on hepatocyte apoptosis as Z-VAD-FMK (Figure 3A-D). Together, these observations suggested that FZHY could effectively inhibit hepatocyte apoptosis both *in vivo and in vitro*. It was well documented that the apoptotic signal induced by Act D plus TNF-α was initially transduced by an extrinsic signaling pathway. TNF-α binds to two cell surface receptors known as TNF-R1 and TNF-R2, with the apoptotic effects of TNF-α being mainly mediated by TNF-R1 [35]. It is well documented that TNF-mediated hepatocyte apoptosis also requires the activation of mitochondria [36]. The increase in mitochondrial membrane permeability is a pivotal event in its activation. This process is regulated by the Bcl-2 family of proteins that are divided into proapoptotic and anti-apoptotic proteins and are best described as mediators of mitochondrial dysfunction [37]. Therefore, we attempted to determine whether FZHY exerted an effect on expressions of TNF-R1, Bcl-2 and Bax. The findings obtained by immunoblotting showed that FZHY and Z-VAD-FMK dramatically downregulated expressions of TNF-R1 and Bax and upregulated Bcl-2 expression.

In the present study, mice injected by CCl_4_ showed a characteristic of massive hepatocytes apoptosis in early stage of liver injury and severe hepatic fibrosis in later stage. Hepatocyte apoptosis, one of the ubiquitous features of acute and chronic liver injuries, is reported to associate with fibrosis tightly. A hepatocyte specific disruption of Bcl-x_L_, an antiapoptotic member of the Bcl-2 family, leads to continuous hepatocyte apoptosis and later liver fibrotic responses. In contrast, inhibition of hepatocyte apoptosis by caspase inhibitor leads to less liver injury and fibrosis [30]. Researchers find that phagocytosis of hepatocyte-derived apoptotic bodies by HSCs is profibrogenic as it induces collagen α1 (I) and TGF-β1 upregulation [38]. DNA extracted from apoptotic hepatcoytes induces differentiation of mouse and human HSCs [4]. These data suggest that inhibiting hepatocyte apoptosis may be one of the mechanisms for attenuating hepatic fibrosis by drugs. The anti-apoptotic effects and mechanisms of FZHY on hepatocytes were well elucidated in both animal and cell models. Meanwhile, we also confirmed that FZHY markedly decreased the expression of α-SMA in the fibrotic liver examined by immunohistochemical staining, as well as attenuated extracellular matrix (ECM) deposition in liver. However, whether or not FZHY prevented HSC activation and hepatic fibrosis through its inhibitory effect on hepatocyte apoptosis? Our results showed that the DNA of apoptotic hepatocytes stimulated HSC activation in a dose dependent manner, and the attenuated hepatocyte apoptosis with FZHY or Z-VAD-FMK treatment reduced HSC activation which reduces ECM deposition. The exact mechanism for this occurrence is not known.

## Conclusions

FZHY suppressed hepatocyte apoptosis elicited by CCl_4_*in vivo* or ActD/TNF-α *in vitro* through regulating mediators in death receptor and mitochondrial pathways, and this action on hepatocyte apoptosis might play an important role on HSC activation and liver fibrosis.
